# Therapeutic Potential of Extracellular Vesicles in Degenerative Diseases of the Intervertebral Disc

**DOI:** 10.3389/fbioe.2020.00311

**Published:** 2020-04-17

**Authors:** Nathan Piazza, Mehdi Dehghani, Thomas R. Gaborski, Karin Wuertz-Kozak

**Affiliations:** ^1^Department of Biomedical Engineering, Rochester Institute of Technology (RIT), Rochester, NY, United States; ^2^Institute for Biomechanics, Zurich, Switzerland; ^3^Spine Center, Schön Clinic Munich Harlaching, Munich, Germany; ^4^Academic Teaching Hospital and Spine Research Institute, Paracelsus Medical University, Salzburg, Austria

**Keywords:** extracellular vesicle, microRNA, back pain, inflammation, regeneration, mesenchymal stem cell, notochordal cell, nucleus pulposus

## Abstract

Extracellular vesicles (EVs) are lipid membrane particles carrying proteins, lipids, DNA, and various types of RNA that are involved in intercellular communication. EVs derived from mesenchymal stem cells (MSCs) have been investigated extensively in many different fields due to their crucial role as regeneration drivers, but research for their use in degenerative diseases of the intervertebral disc (IVD) has only started recently. MSC-derived EVs not only promote extracellular matrix synthesis and proliferation in IVD cells, but also reduce apoptosis and inflammation, hence having multifunctional beneficial effects that seem to be mediated by specific miRNAs (such as miR-233 and miR-21) within the EVs. Aside from MSC-derived EVs, IVD-derived EVs (e.g., stemming from notochordal cells) also have important functions in IVD health and disease. This article will summarize the current knowledge on MSC-derived and IVD-derived EVs and will highlight areas of future research, including the isolation and analysis of EV subpopulations or exposure of MSCs to cues that may enhance the therapeutic potential of released EVs.

## Introduction

Degenerative disc disease (DDD) is a major origin of low back pain, which is the leading cause of activity limitation and work absence and results in a high economic burden (Pai and Sundaram, 2004). In the United States, the yearly costs related to back pain are around 100–200 billion USD (Katz, 2006). DDD is defined as symptomatic intervertebral disc (IVD) degeneration, whereby nociception is thought to be linked to increased levels of proinflammatory cytokines within the tissue, including IL-1β, TNF-α, IL-6, IL-8, and IFN-γ (Wuertz and Haglund, 2013; Johnson et al., 2015). While current treatment options for DDD, including oral analgesics and surgery, solely aim to reduce the symptoms, researchers have made extensive efforts over the past decade to develop novel therapeutic approaches that target the underlying pathophysiological mechanisms, i.e., degeneration, inflammation, and enhanced apoptosis (Fernandez-Moure et al., 2018; Tendulkar et al., 2019). A wide range of approaches has been tested which are described in detail elsewhere (Krupkova et al., 2018; Smith et al., 2018; Clouet et al., 2019; Hodgkinson et al., 2019; Loibl et al., 2019), ranging from autologous disc cell therapy, growth factors, biologics, gene transfection, and biomaterials to CRISPR/Cas9 genome engineering, as well as the use of mesenchymal stem cells (MSCs). However, none of these concepts have been successfully incorporated yet into daily clinical practice.

As in numerous other fields, great hopes were pinned on MSCs for the treatment of DDD due to their regenerative and anti-inflammatory potential that would allow simultaneously targeting all primary pathological drivers (Vadala et al., 2016; Loibl et al., 2019). Furthermore, MSCs have excellent proliferation characteristics and good accessibility from either bone marrow or fat tissue. However, numerous studies demonstrated that the success of MSC-based treatments is limited due to the harsh microenvironment of the degenerated IVD that hampers their survival and functionality (Wuertz et al., 2008; Huang et al., 2013). Although various techniques have recently been developed that may promote MSC survival and functionality in the IVD, such as priming and pre-differentiation of MSCs, selection of specific MSC subpopulations, and rewiring of the genetic circuits of MSCs by CRISPR (Krupkova et al., 2018; Loibl et al., 2019), there has been an increasing shift toward cell-free, yet MSC-related therapies to avoid the aforementioned challenges. While early studies investigated the regenerative and anti-inflammatory potential of MSC-conditioned media in IVD cells (Lv et al., 2014; Teixeira et al., 2018), extracellular vesicles (EVs) have increasingly moved into this area of research. EVs are lipid membrane particles that carry proteins, lipids, DNA, and various types of RNA, and function as nanocarriers with relevance in intercellular communication. As recently summarized by Keshtkar et al. (2018), EVs may be the primary or possibly sole driver of MSC-driven regeneration and could thus represent a potential alternative treatment for DDD. Although very few studies thus far have compared the potential of conditioned media in triggering cell responses to EVs isolated from the same conditioned medium, first evidence suggests a comparable (astrocytes on motor neurons; notochordal cells on chondrocyte-like IVD cells) (Bach et al., 2017; Varcianna et al., 2019) or enhanced beneficial effect (umbilical cord MSCs on T cells) (Monguió-Tortajada et al., 2017) of EVs. However, conclusions are challenging to draw as dose-effects are difficult to control for in these experimental set-ups.

This mini-review will briefly summarize both established and more advanced techniques for the collection and size-sorting of EVs before presenting evidence on the content and function of MSC-derived EVs on cells from the inner region of the IVD, the nucleus pulposus (NP). Furthermore, knowledge on the characterization of EVs released from IVD cells are briefly summarized and future areas of research are highlighted.

## EV Terminology

With a rapid increase in EV-related research over the past years, one of the major limitations is the use of heterogeneous, often field-specific, nomenclature. In IVD research, the term exosome is commonly used, whereas the terms microvesicles, apoptotic bodies, and microparticles are less common. Importantly, the rather historical classification of these particles according to size is outdated as recent studies have shown significant overlap in particle subtype sizes (Kowal et al., 2016; Tkach et al., 2017). Knowledge of particle biogenesis is necessary to accurately identify specific subtypes (Gould and Raposo, 2013; Kowal et al., 2014). Ongoing research may be able to provide specific markers for the various particle subtypes and thus allow accurate classification in the future. Hence, according to the recommendation of the International Society of Extracellular Vesicles (ISEV) and the Minimal Information for Studies of Extracellular Vesicles, 2018 (MISEV2018) (Thery et al., 2018), we will use the name EVs in this mini-review as the generic term for particles naturally released from cells that are delimited by a lipid bilayer and cannot replicate.

## Collection and Purification of EVs

In order to study the biological roles and potential applications of EVs, the first step is to reliably and efficiently isolate them from biological fluids and conditioned media. Ideal isolation methods purify intact EVs with preserved biological function and without contamination by proteins or non-EV particles. Over the past years, many techniques have been developed to isolate EVs based on properties such as size, density, and surface proteins, including ultracentrifugation, ultrafiltration, precipitation, and affinity-based techniques.

The most commonly employed technique was first described by Thery et al. (2006) and is known as differential ultracentrifugation, which refers to a stepwise increase of centrifugal forces that results in sequential pelleting of different size of particles. However, differential ultracentrifugation is a time consuming, expensive process with co-isolation of contaminants and low yields. To increase the purity of the EV sample, differential centrifugation is often coupled with other techniques such as ultrafiltration through 0.2, 0.4, and 0.8 um filters, yet with negative impact on the overall EV yield and the potential for deformation and damage of EVs (Yakimchuk, 2015). Due to aforementioned limitations and complications, a number of research efforts have aimed to develop more reproducible, reliable, and standardized methods that avoid contamination and damage to EVs, including size exclusion chromatography (de Menezes-Neto et al., 2015; Lobb et al., 2015; Gamez-Valero et al., 2016), filtration (Heinemann et al., 2014; Woo et al., 2017; Dehghani et al., 2019), immuno-affinity based approaches (Kabe et al., 2019), and microfluidic techniques (Lee et al., 2015; Zhang P. et al., 2016).

In the IVD field, the majority of studies have used differential centrifugation protocols. Some modified these protocols by adding a filtration step between the centrifugation steps, while others replaced the ultracentrifugation step by the use of commercially available precipitation-based kits (e.g., “Total Exosome Isolation Reagent”). Precipitation-based techniques have a high yield but tend to suffer from low purity. Two IVD studies showed fractionation of conditioned media by ultrafiltration through membranes with different molecular weight cut-offs (MWCO), yielding spherical species with a size range of 50–100 nm. Overall, techniques currently used in IVD research for collection and purification of EVs are widespread and common, but could be replaced by recent technical advancements. By incorporating newer approaches and more sophisticated techniques such as tangential flow filtration (TFF) and size exclusion chromatography (Corso et al., 2017; Mol et al., 2017; Dehghani et al., 2019), reliability and reproducibility of IVD studies could likely be further improved.

## Production, Stability and Storage of EVs

The lipid bilayer membrane of EVs plays an important role in their stability by protecting their bioactive cargo against degradation (Jin et al., 2016). In order to fully utilize EVs for clinical applications, a better understanding of the effect of pH, temperature and storage conditions on their stability is required. Different storage techniques such as cryopreservation (Best, 2015), lyophilization (Kumar et al., 2014), and spray drying have been reported (Kusuma et al., 2018). Storage at −80°C has been recognized as the most promising method for long-term preservation of EV properties and functions. However, it is important to note that studying their stability of EVs can be affected by their cells of origin, purification technique and characterization method (Jeyaram and Jay, 2017). Interestingly, slightly acidic conditions like those of the IVD have been shown to both increase the release as well as uptake of EVs, especially in cancer cells (Parolini et al., 2009; Qi et al., 2016; Cheng et al., 2019).

## Characterization of EVs

The presence of EVs in a purified sample should be confirmed by multiple methods. Western blot (WB) has been employed to characterize the isolated samples by evaluating the presence of EV-enriched markers and the absence of negative markers. Alix, TSG101 and surface associated markers such as Tetraspanins (CD9, CD63, and CD81) have been used as positive markers for EVs (Moen et al., 2017; Cheng et al., 2018), while proteins associated with cell compartments other than endosome or plasma membrane such as Calnexin (endoplasmic reticulum) and GM130 (Golgi) have been used as negative markers (Lu et al., 2017; Vonk et al., 2018; Wei et al., 2019). In addition to protein composition, Transmission electron microscopy (TEM) and Nanoparticle Tracking analysis (NTA) are the two most commonly used technique for studying the morphology (cup-shaped with double layer membrane structures), size and concentration of EVs (Cheng et al., 2018; Liao et al., 2019). In the future, isolation of different subpopulations of EVs based on their biogenesis (exosomes, microvesicles and apoptotic bodies) and size (small, medium and large) are crucial for identifying their therapeutic potential. Moreover, high-resolution flow cytometry can be applied for single EV analysis which provides valuable information regarding size, concentration and identifying the phenotype subsets of EVs.

## Delivery of EVs

Although systemic delivery of EVs is generally considered the easiest approach, biodistribution patterns indicate accumulation in the liver, spleen, and lungs (Di Rocco et al., 2016). Especially when taking the avascular nature of the IVD into account, local delivery has to be regarded as the prime route of administration for future clinical applications. Whether or not EVs should be embedded into biomaterial-based carriers will, however, require further investigation, as does the optimal dose of EVs.

While defining optimal doses has been a crucial aspect in MSC-based IVD therapies due to the limited nutritional supply within the tissue (Loibl et al., 2019), EV dosing will likely be less critical. However, difficulties to obtain large amounts of EVs, combined with the associated high costs, will nonetheless require determining the minimal EV concentration that provides adequate therapeutic effects.

Extracellular vesicles have been shown to bind to ECM-proteins such as fibronectin and collagen type I (Narayanan et al., 2016), which may be sufficient to ensure their captivity within the IVD tissue, despite daily mechanical loading and consequential fluid flow out of the tissue. Should *in vivo* tracking studies of EVs (labeled e.g., by superparamagnetic iron oxide nanoparticles, radioisotopes or fluorescent dyes) (Di Rocco et al., 2016), however, demonstrate high loss of EVs from the IVD, biomaterial-based delivery approaches, similar to those used for MSCs themselves (Bowles and Setton, 2017), may become useful. In fact, a previous study on wound healing has demonstrated that the use of a chitosan/silk hydrogel provided sustained release of EVs while preserving their function (Shi et al., 2017).

## EVs From Ivd Cells: Function and Content

**FIGURE 1:**
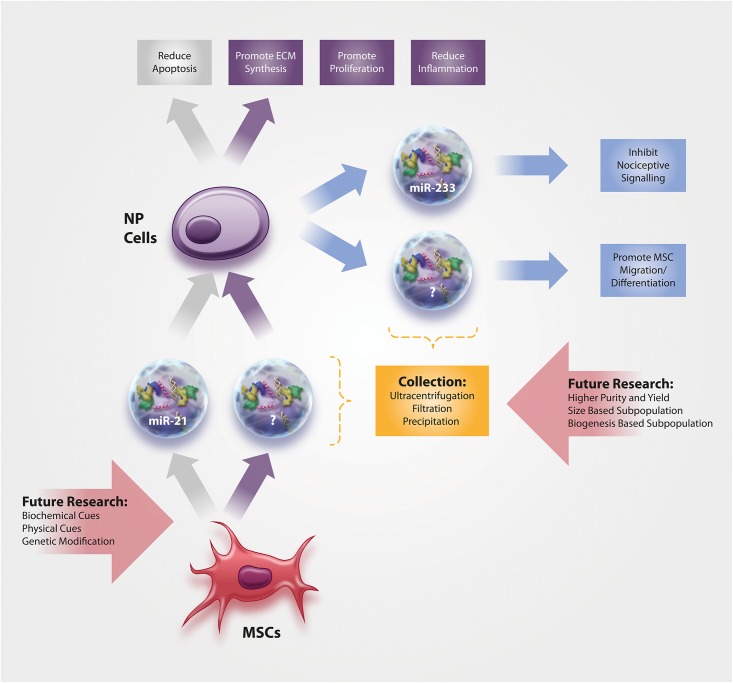
Current knowledge on the function and content of (1) NP-derived EVs and of (2) MSC-derived EVs for the treatment of NP cells. No data exists yet on annulus fibrosus (AF) cells, the more fibroblast-like cells from the outer area of the IVD.

Only few studies have thus far been conducted on IVD-derived EVs, although they most certainly play a crucial role during IVD health and disease. In fact, researchers in other fields have become increasingly interested in the analysis of EVs from resident cells, with the hope to identify specific disease biomarkers (Chen et al., 2018). With the current interest in biomarkers for DDD (Khan et al., 2017; Boisson et al., 2019), research in this context will certainly arise in the short- to mid-term future. Nonetheless, past research has been limited to the function and content of IVD-derived EVs (Figure 1), with a focus on miRNAs as these small non-coding RNA molecules are being extensively investigated for their therapeutic potential in other fields. Park et al. (2019) provide a comprehensive overview of EV-miRNAs with biological relevance in a broad range of application areas, including miR-let-7b, miR-let-7c, miR-17-92, miR-21, miR-23b, miR-125a, miR-133b, miR-146a, miR-221, miR-223, and miR-1587.

Moen et al. (2017) demonstrated that NP cells in a rodent herniation model produce EVs containing miR-223. Importantly, miR-223 was furthermore found to decrease the C-fiber response in the dorsal horn neurons and to inhibit the nociceptive spinal signaling compared to PBS controls, hence indicating its protective role during IVD herniation and chronic lumbar radicular pain. Interestingly, EV-derived miR-223 has also been shown to downregulate inflammation following cardiac surgery with cardiopulmonary bypass (Poon et al., 2017) and this anti-inflammatory function of miR-223 (through modulation of the NF-kB pathway) was already confirmed in NP cells (Wang et al., 2018). However, Wang et al. analyzed the anti-inflammatory role of miR-233 in the IVD in miR-233-overexpressing NP cells (and did not investigate miR-233 in released EVs).

Concerning function of IVD-derived EVs, data on notochordal NP cells and chondrocyte-like NP cells have been published. Notochordal cells are a developmental cell type in the NP that, at least in humans and some other species, gets increasingly replaced by chondrocyte-like IVD cells over the course of life; the reduction in notochordal cell number is believed to be associated with the onset of IVD degeneration (Rodrigues-Pinto et al., 2014). Notochordal cell-derived EVs increased DNA and glycosaminoglycan content in human NP cell micro-aggregates compared to untreated control medium, although the underlying mechanism or the associated EV content was not analyzed in this study by the Tryfonidou Lab (Bach et al., 2017). EVs harvested from human NP cells derived from patients with lumbar degenerative disease were able to promote MSC migration and differentiation into an NP-like phenotype (Lu et al., 2017) through the Notch1 pathway (Lan et al., 2019), although the EV content responsible for this action (e.g., specific miRNAs) remains unknown at this time. However, a delicate interplay between different miRNAs and components of Notch signaling pathway has been identified in cancer, pointing toward miR-146a, miR-19, miR-100, miR-21, miR-181a-1/b-1, miR-375, and miR-483-5p as potentially interesting candidates for further investigation in the IVD field (Majidinia et al., 2018).

## EVs From Mscs: Effects on Ivd Cells and Content

Ample evidence exists from a variety of fields that MSC-derived EVs have therapeutic effects (Park et al., 2019), including musculoskeletal applications (Alcaraz et al., 2019). To ensure reproducibility of results and facilitate the exchange of data among investigators, it is essential that researchers characterize their MSCs according to the guidelines of the International Society for Cellular Therapy (plastic-adherence; lineage differentiation potential; expression of CD105, CD73 and CD90; lack expression of CD45, CD34, CD14 or CD11b, CD79alpha or CD19, and HLA-DR) (Dominici et al., 2006).

As the success of MSC cell therapies for IVD diseases has been hampered due to the restricted survival and functionality of the cells in the harsh IVD microenvironment (Wuertz et al., 2008; Huang et al., 2013), the use of cell-free therapeutic applications may circumvent many of the past translation obstacles, especially if they could halt or reverse the hallmarks of DDD (Figure 1). However, it has to be noted that no studies have thus far directly investigated whether the harsh microenvironment of the IVD might affect EV functionality. Cheng et al. (2019) investigated the stability of EVs derived from HEK 293T cells at pH 4 and pH 7 and found a concentration loss at pH 4, which, however, is too acidic even for the degenerated IVD. On the other hand, acidic microenvironments may actually increase the uptake of EVs (Parolini et al., 2009; Qi et al., 2016; Cheng et al., 2019).

### Preventing Apoptosis and Promoting Proliferation

Cellular loss, predominantly through apoptosis, is an evident hallmark and is thought to play a crucial role in the degenerative processes of the IVD. Therefore, minimizing cell death has been discussed as an auspicious therapeutic strategy for IVD degeneration (Ding et al., 2013). MSC-derived EVs have proven to be effective modulators of NP cell survival and apoptosis, whereby the effect seems to be mediated through activation of the pro-survival PI3K-Akt pathway and ERK pathway, possibly through miR-21 (Cheng et al., 2018; Liao et al., 2019). The apoptosis-reducing effect of EVs via PI3K-Akt and ERK has been confirmed in chondrocytes (Zhang et al., 2018), while the role of miR-21 was confirmed by miR-21 enriched EVs in cardiomyocytes *in vitro* and during myocardial infarction *in vivo* (Song et al., 2019). In the IVD, the inhibition of cell apoptosis through EVs, potentially in combination with other unidentified mechanisms, decelerated progression of IVD degeneration in a rat tail degeneration model based on the injection of advanced glycation end products (Liao et al., 2019).

Aside from attenuating apoptosis, MSC-derived EVs also have the potential to promote proliferation of NP cells (Lu et al., 2017). The mechanisms thereof are thus far unidentified, although AKT and ERK signaling may likely be involved (Pratsinis et al., 2012).

### Inducing ECM Synthesis

A loss of proteoglycans and collagen-II in the NP is one of the most prominent features of IVD degeneration and DDD, clearly visible through MR-based imaging as a black disc. Therefore, regenerative IVD therapies typically focus on promoting the synthesis of IVD-typical extracellular matrix (ECM) proteins by various means, including MSCs and MSC-derived EVs. In fact, Lu et al. (2017) provided compelling evidence that MSC-derived EVs can induce a healthier ECM production in NP cells, as indicated by enhanced gene expression and synthesis of aggrecan, SOX-9 and collagen-II. The efficacy of MSC-derived EVs has also been demonstrated in numerous cartilage repair/regeneration studies (Zhu et al., 2017; Boere et al., 2018; Mao et al., 2018; Tofino-Vian et al., 2018), whereby these effects may be mediated through miR-92a-3p and WNT5A (Mao et al., 2018). Evidently, more studies will be needed to confirm the capacity of EVs to promote IVD health and homeostasis, but also to identify the EV content responsible for the beneficial effects.

### Modulating Inflammation

As chronic inflammation in the IVD arising from resident cells (and in the case of structural failure of the tissue also from invading immune cells) is associated with back pain development (Wuertz and Haglund, 2013; Johnson et al., 2015), many researchers consider the modulation of inflammation as therapeutically more relevant and achievable than inducing full regeneration. Following this strategy, Xia et al. (2019) tested whether MSC-derived EVs can reduce H_2_O_2_-induced inflammation in NP cells. Results of this study provide compelling evidence for anti-inflammatory properties of EVs via modulation of the NLRP3 inflammasome and through restoration of damaged mitochondria (Xia et al., 2019). Although not yet investigated in the IVD, NLRP3 inflammasome activity is known to be controlled by miR-223 (Bauernfeind et al., 2012). Importantly, the positive effects of EVs were not restricted to *in vitro* conditions but could also be replicated in a rabbit IVD degeneration model (Xia et al., 2019). Whether MSC-derived EVs may also affect the phenotype of invaded macrophages in the IVD and thus cause a shift from an inflammatory M1 toward a regenerative M2 phenotype is currently unclear, albeit initial data in that respect could be collected in skin (He et al., 2019).

To further improve the outcome of EV therapy, MSCs can be modified to produce EVs with enhanced or reduced content of certain components with biological activity, such as specific miRNAs. With an increasing understanding of the role of certain miRNAs in IVD health and disease (Zhou et al., 2017), EV therapy may thus be optimized in a tissue- and disease-specific manner. In cartilage for example, miRNA-140 has been identified to regulate tissue homeostasis. Transduction of MSCs with lentiviral miR-140-5p resulted in EVs that increased chondrocyte proliferation and promoted cartilage regeneration compared to the control (= negative control oligonucleotides/vector group) (Wang et al., 2019). Clearly, EVs from modified MSCs have a significant potential as future therapeutic strategies. Similar approaches can be envisioned in the IVD field, targeting for example miR-210, which was shown to inhibit NP cell apoptosis (Zhang D. Y. et al., 2016), or potentially also miR-483, which is downregulated during intervertebral disc disease (Sherafatian et al., 2019).

Despite the promise that initial studies on MSC-derived EVs have sparked, one of the remaining challenges may be the limited distribution of EVs within the IVD due to the densely packed collagen and proteoglycan-rich ECM. However, this dense ECM may also help to retain EVs within the IVD following intradiscal injections. Mathematical models should be employed in the future to further investigate this question and to determine whether specific EV sizes or EV membrane compositions would be best suited for IVD applications. However, these parameters will not only affect tissue retention and distribution, but also biological activity, and both aspects will need to be considered (Margolis and Sadovsky, 2019).

## Discussion and Outlook

First research results on MSC-derived EVs for the treatment of DDD clearly demonstrate their therapeutic potential by promoting regeneration and proliferation and reducing inflammation and apoptosis in the IVD, although the exact mechanisms of action are not yet fully elucidated.

Ample studies in the past have investigated MSC-based therapies for the IVD, leading to increasing knowledge on the bidirectional crosstalk between MSCs and IVD cells. However, MSC cell therapy for the IVD is hampered by the harsh tissue microenvironment; furthermore, cell therapies face safety concerns and thus approval challenges. Although few studies have investigated the effect of e.g., acidic pH on EV stability, it is conceivable that the negative effects that the harsh IVD microenvironment has on MSCs themselves is not relevant for EV-based therapies. In fact, first studies demonstrated a possible increase of cellular uptake of EVs under IVD like pH conditions. Investigating the effect of the harsh IVD microenvironment on EVs is thus an important topic for future investigation.

Despite these advantages of EVs compared to MSC cell therapy, a major limitation lies in their low yields. However, technical advancements related to fast expansion of MSCs as well as enhanced release of EVs that can be induced by commercially available media and additives in combination with new purification strategies will likely help to overcome this restraining factor.

It has become increasingly evident that exposure of MSCs to biophysical and/or biochemical cues may help to further improve the clinical outcome. Therefore, future investigations should aim to identify which signals can enhance the regenerative and/or the anti-inflammatory capacity of EVs, specifically on IVD cells. Alternatively, studies may identify certain subpopulations of EVs that have increased therapeutic properties and/or enhanced tissue retention, based on size characteristics or membrane composition, for example. The use of a biomaterial-based carrier for the *in vivo* delivery of EVs may further enhance the therapeutic benefits through improved captivity within the IVD tissue, sustained release and/or preservation of function.

With the development of novel techniques for the production, collection and delivery of EVs or EV subpopulations, ample possibilities for optimization of EV therapies may arise. However, a better understanding and subsequent optimization of the effects that temperature and storage conditions have on EV stability will be required to promote successful translation into the clinical setting.

## Author Contributions

NP, MD, TG, and KW-K contributed to the literature search, the design of the figure, and the writing of the manuscript.

## Conflict of Interest

The authors declare that the research was conducted in the absence of any commercial or financial relationships that could be construed as a potential conflict of interest.

## References

[B1] Alcaraz M. J. Compañ A. Guillén M. I. (2019). Extracellular vesicles from mesenchymal stem cells as novel treatments for musculoskeletal diseases. *Cells* 9:9010098. 10.3390/cells9010098 31906087 PMC7017209

[B2] Bach F. Libregts S. Creemers L. Meij B. Ito K. Wauben M. (2017). Notochordal-cell derived extracellular vesicles exert regenerative effects on canine and human nucleus pulposus cells. *Oncotarget* 8 88845–88856. 10.18632/oncotarget.21483 29179481 PMC5687651

[B3] Bauernfeind F. Rieger A. Hornung V. (2012). NLRP3 inflammasome activity is negatively controlled by miR-223. *Immunology* 137 292–292. 10.4049/jimmunol.1201516 22984082

[B4] Best B. P. (2015). Cryoprotectant toxicity: facts, issues, and questions. *Rejuvenat. Res.* 18 422–436. 10.1089/rej.2014.1656 25826677 PMC4620521

[B5] Boere J. Malda J. van de Lest C. H. A. van Weeren P. R. Wauben M. H. M. (2018). Extracellular vesicles in joint disease and therapy. *Front. Immunol.* 9:2575. 10.3389/fimmu.2018.02575 30483255 PMC6240615

[B6] Boisson M. Borderie D. Henrotin Y. Teboul-Core S. Lefevre-Colau M. M. Rannou F. (2019). Serum biomarkers in people with chronic low back pain and Modic 1 changes: a case-control study. *Sci. Rep.* 9:10005. 10.1038/s41598-019-46508-x 31292506 PMC6620434

[B7] Bowles R. D. Setton L. A. (2017). Biomaterials for intervertebral disc regeneration and repair. *Biomaterials* 129 54–67. 10.1016/j.biomaterials.2017.03.013 28324865 PMC5627607

[B8] Chen Y. F. Tang Y. L. Fan G. C. Duan D. D. (2018). Extracellular vesicles as novel biomarkers and pharmaceutic targets of diseases. *Acta Pharmacol. Sin.* 39 499–500. 10.1038/aps.2018.15 29606702 PMC5888692

[B9] Cheng X. Zhang G. Zhang L. Hu Y. Zhang K. Sun X. (2018). Mesenchymal stem cells deliver exogenous miR-21 via exosomes to inhibit nucleus pulposus cell apoptosis and reduce intervertebral disc degeneration. *J. Cell Mol. Med.* 22 261–276. 10.1111/jcmm.13316 28805297 PMC5742691

[B10] Cheng Y. Zeng Q. Han Q. Xia W. (2019). Effect of pH, temperature and freezing-thawing on quantity changes and cellular uptake of exosomes. *Protein Cell* 10 295–299. 10.1007/s13238-018-0529-524 29616487 PMC6418301

[B11] Clouet J. Fusellier M. Camus A. Le Visage C. Guicheux J. (2019). Intervertebral disc regeneration: from cell therapy to the development of novel bioinspired endogenous repair strategies. *Adv. Drug Deliv. Rev.* 146 306–324. 10.1016/j.addr.2018.04.017 29705378

[B12] Corso G. Mager I. Lee Y. Gorgens A. Bultema J. Giebel B. (2017). Reproducible and scalable purification of extracellular vesicles using combined bind-elute and size exclusion chromatography. *Sci. Rep.* 7:11561. 10.1038/s41598-017-10646-x 28912498 PMC5599601

[B13] de Menezes-Neto A. Saez M. J. F. Lozano-Ramos I. Segui-Barber J. Martin-Jaular L. Ullate J. M. E. (2015). Size-exclusion chromatography as a stand-alone methodology identifies novel markers in mass spectrometry analyses of plasma-derived vesicles from healthy individuals. *J. Extracell. Vesicles* 4:27378. 10.3402/jev.v4.27378 26154623 PMC4495624

[B14] Dehghani M. Lucas K. Flax J. McGrath J. Gaborski T. (2019). Tangential flow microfluidics for the capture and release of nanoparticles and extracellular vesicles on conventional and ultrathin membranes. *Adv. Mater. Technol.* 4:1900539 10.1002/admt.201900539 PMC7212937 32395607

[B15] Di Rocco G. Baldari S. Toietta G. (2016). Towards therapeutic delivery of extracellular vesicles: strategies for. *Stem Cells Int* 2016:5029619. 10.1155/2016/5029619 27994623 PMC5141304

[B16] Ding F. Shao Z. W. Xiong L. M. (2013). Cell death in intervertebral disc degeneration. *Apoptosis* 18 777–785. 10.1007/s10495-013-0839-831 23512131

[B17] Dominici M. Le Blanc K. Mueller I. Slaper-Cortenbach I. Marini F. Krause D. (2006). Minimal criteria for defining multipotent mesenchymal stromal cells. The International Society for Cellular Therapy position statement. *Cytotherapy* 8 315–317. 10.1080/14653240600855905 16923606

[B18] Fernandez-Moure J. Moore C. A. Kim K. Karim A. Smith K. Barbosa Z. (2018). Novel therapeutic strategies for degenerative disc disease: review of cell biology and intervertebral disc cell therapy. *SAGE Open Med.* 6:2050312118761674. 10.1177/2050312118761674 29568524 PMC5858682

[B19] Gamez-Valero A. Monguio-Tortajada M. Carreras-Planella L. Franquesa M. Beyer K. Borras F. E. (2016). Size-Exclusion Chromatography-based isolation minimally alters Extracellular Vesicles’ characteristics compared to precipitating agents. *Sci. Rep.* 6:33641. 10.1038/srep33641 27640641 PMC5027519

[B20] Gould S. J. Raposo G. (2013). As we wait: coping with an imperfect nomenclature for extracellular vesicles. *J. Extracell. Vesicles* 15:2. 10.3402/jev.v2i0.20389 24009890 PMC3760635

[B21] He X. N. Dong Z. W. Cao Y. N. Wang H. Liu S. Y. Liao L. (2019). MSC-derived exosome promotes M2 polarization and enhances cutaneous wound healing. *Stem Cells Int.* 2019:7132708. 10.1155/2019/7132708 PMC6754952 31582986

[B22] Heinemann M. L. Ilmer M. Silva L. P. Hawke D. H. Recio A. Vorontsova M. A. (2014). Benchtop isolation and characterization of functional exosomes by sequential filtration. *J. Chromatogr. A* 1371 125–135. 10.1016/j.chroma.2014.10.026 25458527

[B23] Hodgkinson T. Shen B. Diwan A. Hoyland J. A. Richardson S. M. (2019). Therapeutic potential of growth differentiation factors in the treatment of degenerative disc diseases. *JOR Spine* 2:e1045. 10.1002/jsp2.1045 31463459 PMC6686806

[B24] Huang Y. C. Leung V. Y. Lu W. W. Luk K. D. (2013). The effects of microenvironment in mesenchymal stem cell-based regeneration of intervertebral disc. *Spine J.* 13 352–362. 10.1016/j.spinee.2012.12.005 23340343

[B25] Jeyaram A. Jay S. M. (2017). Preservation and storage stability of extracellular vesicles for therapeutic applications. *AAPS J.* 20:1. 10.1208/s12248-017-0160-y 29181730 PMC6582961

[B26] Jin Y. Chen K. Wang Z. Wang Y. Liu J. Lin L. (2016). DNA in serum extracellular vesicles is stable under different storage conditions. *BMC Cancer* 16:753 10.1186/s12885-016-2783-2782 PMC5035490 27662833

[B27] Johnson Z. I. Schoepflin Z. R. Choi H. Shapiro I. M. Risbud M. V. (2015). Disc in flames: roles of TNF-alpha and IL-1beta in intervertebral disc degeneration. *Eur. Cell Mater.* 30 104–116. 10.22203/ecm.v030a08 26388614 PMC4751407

[B28] Kabe Y. Sakamoto S. Hatakeyama M. Yamaguchi Y. Suematsu M. Itonaga M. (2019). Application of high-performance magnetic nanobeads to biological sensing devices. *Anal. Bioanal. Chem.* 411 1825–1837. 10.1007/s00216-018-1548-y 30627798 PMC6453870

[B29] Katz J. N. (2006). Lumbar disc disorders and low-back pain: socioeconomic factors and consequences. *J. Bone. Joint. Surg. Am.* 88 (Suppl. 2), 21–24. 10.2106/JBJS.E.01273 16595438

[B30] Keshtkar S. Azarpira N. Ghahremani M. H. (2018). Mesenchymal stem cell-derived extracellular vesicles: novel frontiers in regenerative medicine. *Stem Cell Res. Ther.* 9:63 10.1186/s13287-018-0791-797 PMC5845209 29523213

[B31] Khan A. N. Jacobsen H. E. Khan J. Filippi C. G. Levine M. Lehman R. A. (2017). Inflammatory biomarkers of low back pain and disc degeneration: a review. *Ann. N. Y. Acad. Sci.* 1410 68–84. 10.1111/nyas.13551 29265416 PMC5744892

[B32] Kowal J. Arras G. Colombo M. Jouve M. Morath J. P. Primdal-Bengtson B. (2016). Proteomic comparison defines novel markers to characterize heterogeneous populations of extracellular vesicle subtypes. *Proc. Natl. Acad. Sci. U.S.A.* 113 E968–E977. 10.1073/pnas.1521230113 26858453 PMC4776515

[B33] Kowal J. Tkach M. Thery C. (2014). Biogenesis and secretion of exosomes. *Curr. Opin. Cell Biol.* 29 116–125. 10.1016/j.ceb.2014.05.004 24959705

[B34] Krupkova O. Cambria E. Besse L. Besse A. Bowles R. Wuertz-Kozak K. (2018). The potential of CRISPR/Cas9 genome editing for the study and treatment of intervertebral disc pathologies. *JOR Spine* 1:e1003. 10.1002/jsp2.1003 31463435 PMC6686831

[B35] Kumar V. Qin J. Jiang Y. Duncan R. G. Brigham B. Fishman S. (2014). Shielding of lipid nanoparticles for siRNA delivery: impact on physicochemical properties, cytokine induction, and efficacy. *Mol. Ther. Nucleic Acids* 3:e210. 10.1038/mtna.2014.61 25405467 PMC4459547

[B36] Kusuma G. D. Barabadi M. Tan J. L. Morton D. A. V. Frith J. E. Lim R. (2018) To protect and to preserve: novel preservation strategies for extracellular vesicles. *Front. Pharmacol.* 9:1199 10.3389/fphar.2018.01199 PMC6215815 30420804

[B37] Lan W. R. Pan S. Li H. Y. Sun C. Chang X. Lu K. (2019). Inhibition of the Notch1 pathway promotes the effects of nucleus pulposus cell-derived exosomes on the differentiation of mesenchymal stem cells into nucleus pulposus-like cells in rats. *Stem Cells Int.* 2019:8404168. 10.1155/2019/8404168 PMC6526523 31249601

[B38] Lee K. Shao H. L. Weissleder R. Lee H. (2015). Acoustic purification of extracellular microvesicles. *Acs Nano* 9 2321–2327. 10.1021/nn506538f 25672598 PMC4373978

[B39] Liao Z. Luo R. Li G. Song Y. Zhan S. Zhao K. (2019). Exosomes from mesenchymal stem cells modulate endoplasmic reticulum stress to protect against nucleus pulposus cell death and ameliorate intervertebral disc degeneration in vivo. *Theranostics* 9 4084–4100. 10.7150/thno.33638 31281533 PMC6592170

[B40] Lobb R. J. Becker M. Wen S. W. Wong C. S. F. Wiegmans A. P. Leimgruber A. (2015). Optimized exosome isolation protocol for cell culture supernatant and human plasma. *J. Extracell. Vesicles* 4:27031. 10.3402/jev.v4.27031 26194179 PMC4507751

[B41] Loibl M. Wuertz-Kozak K. Vadala G. Lang S. Fairbank J. Urban J. P. (2019). Controversies in regenerative medicine: should intervertebral disc degeneration be treated with mesenchymal stem cells? *JOR Spine* 2:e1043. 10.1002/jsp2.1043 31463457 PMC6711491

[B42] Lu K. Li H. Y. Yang K. Wu J. L. Cai X. W. Zhou Y. (2017). Exosomes as potential alternatives to stem cell therapy for intervertebral disc degeneration: in-vitro study on exosomes in interaction of nucleus pulposus cells and bone marrow mesenchymal stem cells. *Stem Cell Res. Ther.* 8:108. 10.1186/s13287-017-0563-9 PMC5424403 28486958

[B43] Lv F. S. Zhou L. X. Lu M. M. Chan D. Zheng Z. Cheung K. M. C. (2014). The potential of umbilical cord derived mesenchymal stem cells in intervertebral disc repair. *Glob. Spine J.* 4:1376649 10.1055/s-0034-1376649

[B44] Majidinia M. Darband S. G. Kaviani M. Nabavi S. M. Jahanban-Esfahlan R. Yousefi B. (2018). Cross-regulation between Notch signaling pathway and miRNA machinery in cancer. *DNA Repair.* 66-67 30–41. 10.1016/j.dnarep.2018.04.002 29723707

[B45] Mao G. P. Zhang Z. J. Hu S. Zhang Z. Q. Chang Z. K. Huang Z. Y. (2018). Exosomes derived from miR-92a-3p-overexpressing human mesenchymal stem cells enhance chondrogenesis and suppress cartilage degradation via targeting WNT5A. *Stem Cell Res. Ther.* 9:247. 10.1186/s13287-018-1004-0 PMC6158854 30257711

[B46] Margolis L. Sadovsky Y. (2019). The biology of extracellular vesicles: the known unknowns. *PLoS Biol.* 17:e3000363. 10.1371/journal.pbio.3000363 31318874 PMC6667152

[B47] Moen A. Jacobsen D. Phuyal S. Legfeldt A. Haugen F. Roe C. (2017). MicroRNA-223 demonstrated experimentally in exosome-like vesicles is associated with decreased risk of persistent pain after lumbar disc herniation. *J. Transl. Med.* 15:89 10.1186/s12967-017-1194-1198 PMC5412060 28460630

[B48] Mol E. A. Goumans M. J. Doevendans P. A. Sluijter J. P. G. Vader P. (2017). Higher functionality of extracellular vesicles isolated using size-exclusion chromatography compared to ultracentrifugation. *Nanomed. Nanotechnol. Biol. Med.* 13 2061–2065. 10.1016/j.nano.2017.03.011 28365418

[B49] Monguió-Tortajada M. Roura S. Gálvez-Montón C. Pujal J. M. Aran G. Sanjurjo L. (2017). Nanosized UCMSC-derived extracellular vesicles but not conditioned medium exclusively inhibit the inflammatory response of stimulated T cells: implications for nanomedicine. *Theranostics* 7 270–284. 10.7150/thno.16154 28042333 PMC5197063

[B50] Narayanan R. Huang C. C. Ravindran S. (2016). Hijacking the cellular mail: exosome mediated differentiation of mesenchymal stem cells. *Stem Cells Int.* 2016:3808674. 10.1155/2016/3808674 26880957 PMC4736778

[B51] Pai S. Sundaram L. J. (2004). Low back pain: an economic assessment in the United States. *Orthop. Clin. North Am.* 35 1–5. 10.1016/S0030-5898(03)00101-109 15062712

[B52] Park K. S. Bandeira E. Shelke G. V. Lasser C. Lotvall J. (2019). Enhancement of therapeutic potential of mesenchymal stem cell-derived extracellular vesicles. *Stem Cell Res. Ther.* 10:288 10.1186/s13287-019-1398-1393 PMC6757418 31547882

[B53] Parolini I. Federici C. Raggi C. Lugini L. Palleschi S. De Milito A. (2009). Microenvironmental pH is a key factor for exosome traffic in tumor cells. *J. Biol. Chem.* 284 34211–34222. 10.1074/jbc.M109.041152 19801663 PMC2797191

[B54] Poon K. S. Palanisamy K. Chang S. S. Sun K. T. Chen K. B. Li P. C. (2017). Plasma exosomal miR-223 expression regulates inflammatory responses during cardiac surgery with cardiopulmonary bypass. *Sci. Rep.* 7:10807. 10.1038/s41598-017-09709-w 28883474 PMC5589826

[B55] Pratsinis H. Constantinou V. Pavlakis K. Sapkas G. Kletsas D. (2012). Exogenous and autocrine growth factors stimulate human intervertebral disc cell proliferation via the ERK and Akt pathways. *J. Orthop. Res.* 30 958–964. 10.1002/jor.22017 22105580

[B56] Qi H. Liu C. Long L. Ren Y. Zhang S. Chang X. (2016). Blood exosomes endowed with magnetic and targeting properties for cancer therapy. *ACS Nano* 10 3323–3333. 10.1021/acsnano.5b06939 26938862

[B57] Rodrigues-Pinto R. Richardson S. M. Hoyland J. A. (2014). An understanding of intervertebral disc development, maturation and cell phenotype provides clues to direct cell-based tissue regeneration therapies for disc degeneration. *Eur. Spine J.* 23 1803–1814. 10.1007/s00586-014-3305-z 24777668

[B58] Sherafatian M. Abdollahpour H. R. Ghaffarpasand F. Yaghmaei S. Azadegan M. Heidari M. (2019). MicroRNA expression profiles, target genes, and pathways in intervertebral disk degeneration: a meta-analysis of 3 microarray studies. *World Neurosurg.* 126 389–397. 10.1016/j.wneu.2019.03.120 30904808

[B59] Shi Q. Qian Z. Liu D. Sun J. Wang X. Liu H. (2017). GMSC-derived exosomes combined with a chitosan/silk hydrogel sponge accelerates wound healing in a diabetic rat skin defect model. *Front. Physiol.* 8:904 10.3389/fphys.2017.00904 PMC5681946 29163228

[B60] Smith L. J. Silverman L. Sakai D. Le Maitre C. L. Mauck R. L. Malhotra N. R. (2018). Advancing cell therapies for intervertebral disc regeneration from the lab to the clinic: recommendations of the ORS spine section. *JOR Spine* 1:e1036. 10.1002/jsp2.1036 30895277 PMC6419951

[B61] Song Y. Zhang C. Zhang J. Jiao Z. Dong N. Wang G. (2019). Localized injection of miRNA-21-enriched extracellular vesicles effectively restores cardiac function after myocardial infarction. *Theranostics* 9 2346–2360. 10.7150/thno.29945 31149048 PMC6531307

[B62] Teixeira G. Q. Pereira C. L. Ferreira J. R. Maia A. F. Gomez-Lazaro M. Barbosa M. A. (2018). Immunomodulation of Human Mesenchymal Stem/Stromal Cells in Intervertebral Disc Degeneration: insights From a Proinflammatory/Degenerative Ex Vivo Model. *Spine* 43 E673–E682. 10.1097/BRS.0000000000002494 29189572

[B63] Tendulkar G. Chen T. Ehnert S. Kaps H. P. Nussler A. K. (2019). Intervertebral disc nucleus repair: hype or hope? *Int. J. Mol. Sci.* 20:3622. 10.3390/ijms20153622 31344903 PMC6696292

[B64] Thery C. Amigorena S. Raposo G. Clayton A. (2006). Isolation and characterization of exosomes from cell culture supernatants and biological fluids. *Curr. Protoc. Cell Biol.* Chapter 3:Unit 322. 10.1002/0471143030.cb0322s30 18228490

[B65] Thery C. Witwer K. W. Aikawa E. Alcaraz M. J. Anderson J. D. Andriantsitohaina R. (2018). Minimal information for studies of extracellular vesicles 2018 (MISEV2018): a position statement of the International Society for Extracellular Vesicles and update of the MISEV2014 guidelines. *J. Extracell. Vesicles* 7:1535750. 10.1080/20013078.2018.1535750 30637094 PMC6322352

[B66] Tkach M. Kowal J. Zucchetti A. E. Enserink L. Jouve M. Lankar D. (2017). Qualitative differences in T-cell activation by dendritic cell-derived extracellular vesicle subtypes. *EMBO J.* 36 3012–3028. 10.15252/embj.201696003 28923825 PMC5641679

[B67] Tofino-Vian M. Guillen M. I. del Caz M. D. P. Silvestre A. Alcaraz M. J. (2018). Microvesicles from human adipose tissue-derived mesenchymal stem cells as a new protective strategy in osteoarthritic chondrocytes. *Cell Physiol. Biochem.* 47 11–25. 10.1159/000489739 29763932

[B68] Vadala G. Russo F. Ambrosio L. Papalia R. Denaro V. (2016). Mesenchymal stem cells for intervertebral disc regeneration. *J. Biol. Regul. Homeost. Agents* 30(4 Suppl. 1), 173–179. 28002916

[B69] Varcianna A. Myszczynska M. A. Castelli L. M. O’Neill B. Kim Y. Talbot J. (2019). Micro-RNAs secreted through astrocyte-derived extracellular vesicles cause neuronal network degeneration in C9orf72 ALS. *EBiomedicine* 40 626–635. 10.1016/j.ebiom.2018.11.067 30711519 PMC6413467

[B70] Vonk L. A. van Dooremalen S. F. J. Liv N. Klumperman J. Coffer P. J. Saris D. B. F. (2018). Mesenchymal stromal/stem cell-derived extracellular vesicles promote human cartilage regeneration. *Theranostics* 8 906–920. 10.7150/thno.20746 29463990 PMC5817101

[B71] Wang H. Hao P. Zhang H. Xu C. Zhao J. (2018). MicroRNA-223 inhibits lipopolysaccharide-induced inflammatory response by directly targeting Irak1 in the nucleus pulposus cells of intervertebral disc. *IUBMB Life* 70 479–490. 10.1002/iub.1747 29707878

[B72] Wang Z. Hu J. L. Pan Y. Shan Y. J. Jiang L. Q. Qi X. (2019). miR-140-5p/miR-149 affects chondrocyte proliferation, apoptosis, and autophagy by targeting FUT1 in Osteoarthritis (vol 41, pg 959, 2018). *Inflammation* 42 1515–1516. 10.1007/s10753-019-01001-5 30903546

[B73] Wei Y. Tang C. Zhang J. Li Z. Zhang X. Miron R. J. (2019). Extracellular vesicles derived from the mid-to-late stage of osteoblast differentiation markedly enhance osteogenesis in vitro and in vivo. *Biochem. Biophys. Res. Commun.* 514 252–258. 10.1016/j.bbrc.2019.04.029 31029430

[B74] Woo H. K. Sunkara V. Park J. Kim T. H. Han J. R. Kim C. J. (2017). Exodisc for rapid, size-selective, and efficient isolation and analysis of nanoscale extracellular vesicles from biological samples. *Acs Nano* 11 1360–1370. 10.1021/acsnano.6b06131 28068467

[B75] Wuertz K. Godburn K. Neidlinger-Wilke C. Urban J. Iatridis J. C. (2008). Behavior of mesenchymal stem cells in the chemical microenvironment of the intervertebral disc. *Spine* 33 1843–1849. 10.1097/BRS.0b013e31817b8f53 18670337 PMC2567058

[B76] Wuertz K. Haglund L. (2013). Inflammatory mediators in intervertebral disk degeneration and discogenic pain. *Glob. Spine J.* 3 175–184. 10.1055/s-0033-1347299 24436868 PMC3854585

[B77] Xia C. Zeng Z. Fang B. Tao M. Gu C. Zheng L. (2019). Mesenchymal stem cell-derived exosomes ameliorate intervertebral disc degeneration via anti-oxidant and anti-inflammatory effects. *Free Radic. Biol. Med.* 143 1–15. 10.1016/j.freeradbiomed.2019.07.026 31351174

[B78] Yakimchuk K. (2015). Exosomes: isolation and characterization methods and specific markers. *Mater. Methods* 5:1450 10.13070/mm.en.5.1450

[B79] Zhang D. Y. Wang Z. J. Yu Y. B. Zhang Y. Zhang X. X. (2016). Role of microRNA-210 in human intervertebral disc degeneration. *Exp. Ther. Med.* 11 2349–2354. 10.3892/etm.2016.3176 27284319 PMC4887766

[B80] Zhang P. He M. Zeng Y. (2016). Ultrasensitive microfluidic analysis of circulating exosomes using a nanostructured graphene oxide/polydopamine coating. *Lab Chip* 16 3033–3042. 10.1039/c6lc00279j 27045543 PMC4970962

[B81] Zhang S. Chuah S. J. Lai R. C. Hui J. H. P. Lim S. K. Toh W. S. (2018). MSC exosomes mediate cartilage repair by enhancing proliferation, attenuating apoptosis and modulating immune reactivity. *Biomaterials* 156 16–27. 10.1016/j.biomaterials.2017.11.028 29182933

[B82] Zhou X. Y. Chen L. L. Grad S. Alini M. Pan H. B. Yang D. Z. (2017). The roles and perspectives of microRNAs as biomarkers for intervertebral disc degeneration. *J. Tissue Eng. Regen. Med.* 11 3481–3487. 10.1002/term.2261 28256798

[B83] Zhu Y. Wang Y. Zhao B. Niu X. Hu B. Li Q. (2017). Comparison of exosomes secreted by induced pluripotent stem cell-derived mesenchymal stem cells and synovial membrane-derived mesenchymal stem cells for the treatment of osteoarthritis. *Stem Cell Res. Ther.* 8:64 10.1186/s13287-017-0510-519 PMC5345222 28279188

